# African Swine Fever Virus Immunosuppression and Virulence-Related Gene

**DOI:** 10.3390/cimb46080488

**Published:** 2024-07-31

**Authors:** Tao Huang, Fangtao Li, Yingju Xia, Junjie Zhao, Yuanyuan Zhu, Yebing Liu, Yingjuan Qian, Xingqi Zou

**Affiliations:** 1 China/WOAH Reference Laboratory for Classical Swine Fever, China Institute of Veterinary Drug Control, Beijing 100081, China; erbercom@163.com (T.H.); 13021967789@163.com (F.L.);; 2 College of Veterinary Medicine, Nanjing Agricultural University, Nanjing 210095, China

**Keywords:** ASFV, immunosuppression, virulence-related genes, pathways

## Abstract

African swine fever virus (ASFV), a highly contagious pathogen characterized by a complex structure and a variety of immunosuppression proteins, causes hemorrhagic, acute, and aggressive infectious disease that severely injures the pork products and industry. However, there is no effective vaccine or treatment. The main reasons are not only the complex mechanisms that lead to immunosuppression but also the unknown functions of various proteins. This review summarizes the interaction between ASFV and the host immune system, along with the involvement of virulence-related genes and proteins, as well as the corresponding molecular mechanism of immunosuppression of ASFV, encompassing pathways such as cGAS-STING, nuclear factor kappa–light-chain-enhancer of activated B cells (NF-κB), Janus Kinase (JAK) and JAK Signal Transducers and Activators of Transcription (STAT), apoptosis, and other modulation. The aim is to summarize the dynamic process during ASFV infection and entry into the host cell, provide a rational insight into development of a vaccine, and provide a better clear knowledge of how ASFV impacts the host.

## 1. Introduction

African swine fever virus (ASFV), the pathogeny of African swine fever (ASF), is a 200 nm diameter double-stranded DNA virus in the family *Asfarviridae*. The viral genome is a linear 170–190 kb and encodes 150–200 viral proteins, but understanding about these proteins is not exactly clear [1,2,3]. The virion exhibits icosahedral symmetry, composed of nucleoid, core shell, inner envelope, capsid, and outer envelope [4]. The capsid proteins account for approximately one third of the total protein of the virus. Surrounding the capsid is the inner envelope derived from the endoplasmic reticulum, which incorporates several significant immune-associated proteins [5,6].

ASFV induces severe clinical symptoms, including fever, redness, viremia, and diarrhea. According to these symptoms, ASF can be divided into three forms, acute, subacute, or chronic [7]. The acute form of ASFV is characterized by a rapid death during the appearance of clinical signs without heavy organ lesion [8]. Infected pigs present diarrhea with blood and organ hemorrhage. Pregnant sows may exhibit miscarriage and stillbirth [9]. The subacute infectious pigs can persist for even 70 days, showing the above symptoms similar to those of acute cases around 6–7 days post-infection; then, some recover health while others experience mild anorexia. Mortality in subacute infectious pigs ranges from 30 to 70% [10]. The chronic infection may last for 2–15 months. During this period, pigs develop fluctuating fever, anorexia, joint swelling, coughing, diarrhea, occasional vomiting, and skin necrosis. The clinical manifestations of ASF closely resemble those of classical swine fever and are also akin to those exhibited in other swine diseases [11]. ASFV-induced outbreaks result in severe adverse economic consequences in affected regions and pose a significant threat to global swine trade due to almost complete mortality and morbidity in susceptible populations [12].

The virus must experience six steps for successfully infecting host cells: adsorption, penetration, uncoating, replication, packaging, and shedding. Upon cellular entry, ASFV sheds its outer membrane and undergoes internalization within endosomes. Subsequent to induction by low pH conditions, fusion between the virus inner membrane and endosomal membrane occurs, leading to the release of the viral core into the cytoplasm [13]. The core is then transported around the nucleus via microtubules and employs its encoded enzymes and cellular factors for early mRNA transcription, translation, and genome replication [14]. The initiation ASFV genome DNA replication commences approximately 5–10 h post-infection of the host cell with the early gene expression. The replication occurs in two stages and its mechanism is similar to that of poxviruses. Initially, a brief replication stage takes place in the nucleus, followed by the synthesis of numerous DNA fragments within the virus factory (VF) located in the perinuclear region. Subsequently, the virus particles undergo assembly in the VF [15]. After replication, many intermediate and late genes express proteins that play a significant role in virus particle assembly and shedding, such as pC962R, pG1211R, pE301R, pE165R, F1055L, and pP1192R, but their exact functions remain barely known [16].

Macrophages are one of the antigen-presenting cells (APCs) and the target cells for ASFV infection and the receptor-mediated endocytosis and clathrin-dependent endocytic pathway are important pathways for ASFV to invade macrophages [17]. While the innate immune response and adaptive immune response are induced, the natural killer cells and dendritic cells are capable of generating significant amounts of interferon to combat ASFV infection, following 8 days of ASFV infection, and then the antibody levels progressively rise, indicating the presence of a humoral immune response in the host. Some studies have reported that specific antibodies are sufficient to protect pigs from lethal challenges of ASFV, but our knowledge is limited for those antibodies [18,19]. Furthermore, cellular immunity, exemplified by CD8a (+) T cells, is crucial in the antiviral defense against ASFV infection. CD4 (+) CD8 (+) double-positive (DP) T cells have the capacity to release perforin and granzyme, which could also contribute to the resistance against ASFV infection [20]. Despite the numerous mechanisms the host immune system possesses to inhibit ASFV invasion, the efficacy is unsatisfactory, as the virus always can easily escape from capture by the host immune system and even suppress it. This review highlights key virulence-associated genes and proteins of ASFV, while also providing an overview of the documented mechanisms for how ASFV influences the host’s innate and adaptive immune responses through regulating the various upstream or downstream signaling factors of signaling pathways and other regulatory processes. It is these intricate mechanisms that elucidate the considerable challenge in developing an effective vaccine.

## 2. ASFV Virulence-Associated Genes and Key Proteins

The ASFV genome consists of the central conserved region, approximately 125 kb in length, flanked by the variable left region (38–48 kb) and right region (13–22 kb). These variable regions contain five unique multigene families (MGFs), *MGF100*, *MGF110*, *MGF300*, *MGF360*, and *MGF530/505*, that exhibit substantial diversity among strains from different sources. Their variability is linked to viral virulence, antigenic variation, and evasion of host immune responses [6] and, especially, the *MGF360* and *MGF505* are responsible for enhancing infected cell survival and influencing the immune response [21]. Currently, we know that ASFV encodes approximately 50 proteins, including structural proteins of the virus, proteins involved in viral particle assembly, enzymes, and factors required for nucleic acid metabolism, replication, repair, transcription, and processing [22]. The immunosuppression proteins encoded by ASFV are mainly categorized by function as follows: (1) regulation of the host cell protein expression system and transcription, such as *DP71L* and *A238L*, blocking protein expression of host cells; (2) inhibition of the type I interferon signaling pathway, including multigene family proteins *MGF360*, *MGF505/530*, *DP96R*, and *I329L*, suppressing the induction of type I interferon; (3) regulation of programmed cell death, including p54, *A179L*, *A224L*, and *EP153R*, hindering apoptosis in the early stage of infection; and (4) other immunosuppressive proteins, such as CD2v and *L83L*, which are the obstacles for lymphocyte proliferation and antiviral effects of IL-1β [23,24]. Additionally, there are still some proteins with functions that are not yet clear (Table 1).

## 3. ASFV Interaction with Host Immune System

The host immune system recognizes and targets ASFV for elimination, so interaction between ASFV and the host immune system is a crucial aspect in understanding the pathogenesis of ASF. The co-ordinated efforts of innate and adaptive immune responses are essential for providing protection against a wide range of pathogens. Type I interferons play a key role in priming immune cells for antiviral defense, while effector cells like macrophages and dendritic cells serve as frontline defenders in detecting and combating invading pathogens. This collaborative action ensures a robust and effective immune response to safeguard the body from infections. The analysis of the macrophage transcriptome identified a suppression of immune regulation involving 54 cytokine genes and reduced expression of 13 cytokines [37]. However, ASFV has developed sophisticated mechanisms to evade and manipulate the host immune response by modulating the above cytokines and other unknown targets, allowing it to establish infection and cause disease in pigs.

### 3.1. ASFV Regulates Innate Immunity

Innate immunity is the body’s first line of defense against pathogens and other foreign invaders. It is a nonspecific immune response that provides immediate protection upon exposure to a threat. APCs detect pathogen-associated molecular patterns (PAMP) through a series of pattern recognition receptors (PRR), including Toll-like receptors, nucleotide oligomerization domain (NOD)-like receptors, and C-type lectin-like receptors, and produce cytokines and chemokines that help in clearing pathogens through phagocytosis. In order to avoid this progress, a multitude of proteins encoded by ASFV genome serve as pivotal regulators in these evasion strategies. For example, *E120R*, a protein encoded by ASFV, can combine with IRF3 and inhibit the activity of IRF3, resulting in the suppression of IFN-β expression [38]; *QP383R*, an inhibitor of activation of type I interferons, suppresses cGAS dimerization by replacing the dsDNA to interact with cGAS, thus leading to a reduction in cGAMP production [39]; *DP96R*, one of the virulence-related genes, can inhibit the activation of the promotor of TBK1 and reduce the level of phosphorylation of TBK1, resulting in the inhibition of Type I interferons and TBK1-induced antiviral effect [40]; *MGF360-13L* has been identified as the pathogenicity gene that antagonizes the production of Type I interferons and inhibits its mediated signal transduction [41]; *I267L*, a highly conserved protein among different ASFV strains, effectively impairs Riplet-mediated RIG-I activation by interacting with Riplet and hindering its association with RIG-I. This disruption interferes with Riplet-mediated K63-linked polyubiquitination of RIG-I, a process where ubiquitin molecules are attached to specific proteins after they have been synthesized, and the recruitment of MAVS, the downstream adaptor protein. By inhibiting the activation of the IFN-β promoter triggered by RIG-I and Riplet, *I267L* hampers the immune response. Moreover, *I267L* negates Riplet-induced polyubiquitination and the activation of RIG-I, potentially aiding ASFV in evading the host’s innate immunity and enhancing virulence [42]. A part of the specific mechanism will be detailed in the following sections.

### 3.2. Modulation with Adaptive Immunity

Upon encountering a pathogen, antigen-presenting cells, like dendritic cells, capture and present antigens to T cells through major histocompatibility complex (MHC) molecules, initiating an adaptive immune response. T cells differentiate into effector T cells that can directly kill infected cells or help activate other immune cells. B cells produce antibodies that bind to and neutralize pathogens, marking them for destruction by other immune cells. Nevertheless, certain ASFV strains inhibit MHC-II-mediated antigen processing, activation of M1 macrophages, or macrophage autophagy and apoptosis pathway [40]. Some studies demonstrated that some ASFV strains interfere with the normal expression of MHC-I and ASFV infection also results in the reduced surface expression of CD14 and CD16 on macrophages, potentially compromising their antimicrobial and antiviral capabilities. This finding implies that ASFV may develop strategies to interfere with macrophage function as an approach to evade the host’s immune response [3]. It has been reported that the infected host shows decreased levels of various CD T cells that are essential in mounting the cellular immune defense against ASFV. Furthermore, a reduction in the total count of CD4 (+) T cells in the peripheral blood of infected pigs was detected [43]. Due to a plunge in these effector cells, the purpose of immune evasion is achieved by ASFV. For example, the *A179L* protein can inhibit sensitized T-cell-induced cell apoptosis by binding to the BH3 domain of the Bcl-2 protein family through a conserved ligand [36]. The ASFV protein DP71L inhibits transcription factor 4 (ATF4) and its downstream target CHOP, thereby suppressing sensitized T-cell-induced cell apoptosis [44]. It recruits PP1 to dephosphorylate eIF2 to promote ASFV replication within the cells [45]. 

## 4. ASFV Immunosuppression Mechanisms

**Figure 1:**
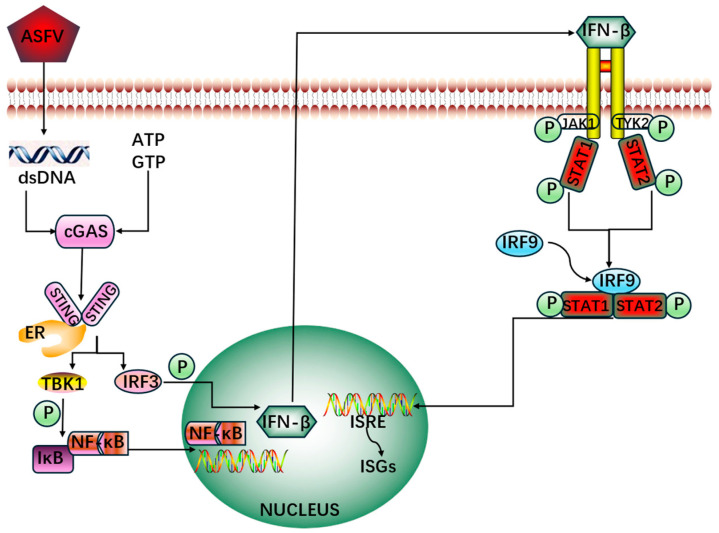
The relations among cGAS-STING, NF-κB, and JAK/STAT signaling pathways. After invasion of ASFV into host cells, the dsDNA of ASFV binds to and activates cGAS, along with the cGAS-STING pathway, and TBKI is activated to phosphorylate IRF3 and initiate the NF-κB pathway, resulting in production of type I interferons. These type I interferons then activate and phosphorylate JAK1 and tyrosine kinase 2, subsequently phosphorylating STAT1 and STAT2. The phosphorylated STAT1 and STAT2 then bind to IRF9 to form the IFN-stimulated gene factor (ISGF) 3 complex; the ISGF3 complex can translocate into the nucleus and boost the activity of IFN-stimulated response element (ISRE), thereby increasing the expression of IFN-stimulated genes (ISGs), which play important roles in the cell’s response to viruses and pathogens. During the above processes, the ASFV is able to influence the normal function of various regulatory factors and promotes its pathogenicity.

ASFV invasion is characterized by immunosuppression; it regulates various signaling pathways to promote viral replication, such as cGAS-STING, nuclear factor kappa–light-chain-enhancer of activated B cells (NF-κB), the Janus Kinase (JAK) and JAK Signal Transducers and Activators of Transcription (STAT), ubiquitination, and apoptosis. These signaling pathways are necessary for the host to bridge the immune system and pathogens so that the host can resist these pathogens. They involve cascading reactions containing numerous regulatory factors, and nodes are significantly influenced by ASFV via phosphorylation or dephosphorylation, degradation or undegradation, and other mechanisms to inhibit the normal function of the host immune system, thus inducing immunosuppression (Figure 1). The specific details are as follows.

### 4.1. Cyclic GMP-AMP Synthase Signaling Pathway

The cGAS/STING signaling pathway plays a crucial role as a key immune regulator in response to pathogens [46]. Extensive research has focused on cGAS’s ability to detect cytosolic or viral DNA. Monomeric cGAS is normally found in the cytoplasm of healthy cells where it cannot bind to DNA or function as an enzyme [47]. However, once cGAS binds to the pathogen’s dsDNA, it triggers the production of cyclic GMP-AMP (cGAMP). This cGAMP molecule then acts as a second messenger by activating the STING adaptor protein, which recruits a series of signaling events involving TBK1 and IRF3 [48], ultimately resulting in their translocation to the nucleus [49]. Activation of this pathway induces the production of type I interferons and proinflammatory cytokines, promoting an effective antiviral immune response [38].

A part of the proteins of ASFV are able to inhibit the expression of type I interferons and antagonize their antiviral effect, which allows ASFV to induce immunosuppression. Overexpression of *QP383R* inhibited the activation of type I interferons triggered by dsDNA and cGAS/STING pathways. *QP383R* was shown to directly interact with cGAS, interfering with its DNA binding ability and dimerization process. This interaction results in the inhibition of cGAS enzymatic functions, ultimately reducing the production of cGAMP, a critical signaling molecule involved in the host immune response against viral infections [50]. ASFV *DP96R*, a kind of conserved early expressed protein, suppresses the activation of the promoter of IFN-β and ISRE, mediated by GAS-STING and phosphorylation of TBK1 [51]. In the *MGF360* multi-gene family, *A276R* has been identified to suppress the production and induction of type I interferon through the IRF3 signaling pathway [52]. Similarly, *A528R* from *MGF505* has been shown to inhibit the type I interferon signaling pathway [53]. Furthermore, deletion of *MGF360-18R* (*DP148R*) results in reduced viral virulence without affecting viral replication in porcine alveolar macrophages (PAM) [54]. *MGF360-13L* inhibits the activation of the cGAS-STING-mediated IFN signaling pathway. It disrupts the assembly of the STING–TBK1–IRF3 complex, leading to the inhibition of TBK1 and IRF3 phosphorylation and dimerization. Ultimately, this restriction limits the nuclear translocation of IRF3 [41].

Gene deletion is a promising approach for developing effective attenuated vaccines, especially after elucidating the specific mechanism of a gene. In order to explore how various genes influence type I IFN via the signaling pathway in vivo, the *MGF505-2R* gene, an ASFV immunomodulator gene involved in controlling the innate immune response through regulation of the cGAS/STING pathway, was deleted from wild-type ASFV. The result showed that the recombinant *MGF505-2R* deletion virus demonstrated characteristics of a potential live attenuated vaccine (LAV), providing approximately 60–70% protection, and may serve as a strong candidate for developing enhanced LAVs, because it lost its ability to interact with STING and inhibit the phosphorylation of TBK1.

### 4.2. NF-κB Signaling Pathway

The NF-κB pathway is a critical signaling pathway involved in the regulation of immune responses, inflammation, cell proliferation, and survival [55]. Upon activation by corresponding factors, the IκB proteins are phosphorylated by the IκB kinase (IKK) complex, releasing NF-κB dimers (typically p50 and RelA or p65) into the nucleus to regulate immune reaction [56]. The ASFV gene-encoded proteins have the capacity of influencing this signaling pathway [57].

Recently, studies have demonstrated that *MGF360-12L*, belonging to the member of ASFV MGF that encodes the most effective antagonist for host immunity defense, can inhibit the production of type I interferons by interacting with KPNA2, KPNA3, and KPNA4, thereby preventing the interaction between importinα and the NF-κB signaling pathway [58]. pMGF505-7R, also encoded by ASFV MGF, inhibits NF-κB activity by binding to IKK, blocking its translocation to the nucleus [59]. This inhibition prevents the phosphorylation of NF-κB p65 and IκB proteins, subsequently leading to the suppression of IL-1β production [60]. The homolog IkBα A238L shares a similar Ankyrin repeat sequence with IkBα. The modified form of A238L interacts directly with the subunit of NF-κB, forming the A238L–p65 complex in the cytoplasm. This complex inhibits the entry of NF-κB p65 into the nucleus and its binding to DNA, thereby resulting in the inhibition of NF-κB pathway [61]. UBCv1, which is the only known conjugating enzyme encoded by the ASFV, has been recognized as a novel inhibitor of NF-κB activation that impedes the movement of p65 into the nucleus [62]. F317L is a protein of ASFV that consists of 317 amino acids and can combine with IκB kinase β (IKKβ) and prevent its own phosphorylating so that it limits the level of IκB ubiquitination and phosphorylation, thereby increasing the IκB stability and suppressing the host immune system, but not all proteins express inhibitive function; *K205R* and *A224L*, two exceptions, have been discovered to contribute P65 to translocate into the nucleus, resulting in the activation of NF-κB [63,64].

The activated NF-κB signaling pathway initiates the release of immune-associated molecules, including IFNs and proinflammatory cytokines. Previous studies have elucidated the specific inhibitory mechanism of *MGF300-4L* on this signaling pathway. Moreover, upon deletion of this gene to investigate its function in vivo, it was observed that the recombinant virus induced a greater proinflammatory response in pigs when compared to wild-type ASFV. Additionally, they also observed that it was capable of eliciting a faster humoral and cellular immune response 

### 4.3. JAK/STAT Signaling Pathway

JAK/STAT signaling pathway is a crucial intracellular signaling cascade that plays a significant role in immune response. Once the activated kinases, JAK and tyrosine kinase2 (TYK2), phosphorylate STAT1 and STAT2, the phosphorylated proteins subsequently form a complex with IFN Regulatory Factor 9 (IRF9), creating the Interferon-Stimulated Gene Factor (ISGF) 3. Finally, the ISGF3 induces a series of signaling activation to initiate the host’s antiviral defense mechanisms [65].

Protein degradation serves as a key mechanism for modulating protein function within biological processes, and the primary strategies employed for protein degradation and regulation include the ubiquitin–proteasome system, autophagy-lysosomal pathway, and apoptosis pathways. *MGF-360-9L*, a virulence factor of the ASFV, binds to and degrades STAT1 and STAT2 through apoptosis and ubiquitin-proteasome pathways, leading to the suppression of IFN-β signaling [66]. *MGF360-10L* specifically interacts with JAK1, resulting in a notable rise in its ubiquitination levels in both HEK293T cells and PAMs. This elevates ubiquitination then triggers the degradation of JAK1 through the proteasome-mediated pathway. Subsequently, through RNA-seq analysis, *MGF-360-10L* was identified to facilitate the ubiquitination of JAK1 by recruiting HERC5 [67]. The CD2v contributes to STAT3 transcription and translocation into the nucleus and prevents the apoptosis of JAK2-STAT3 pathway to facilitate viral replication [25]. In another experiment, researchers found that overexpression of *MGF360-12L* was shown to reduce the expression of IRF9 while not impacting the levels of STAT1 and STAT2. Their analysis of truncated mutations revealed that the C-terminal region of *MGF360-12L* was responsible for suppressing immune responses related to TBK1 and IKKβ. Furthermore, complete sequences of *MGF360-12L* might be necessary for its inhibitory effects on IRF3-5D and IRF9 [68].

Due to the importance of JAK/STAT signaling pathway in vivo and the antagonism of *MGF360-9L* on this signaling pathway, some researchers have found that deletion of MGF-360-10L from wild-type ASFV infection causes milder pathological injury in pigs and weakens the virulence compared to ASFV-WT. Thus, the recombinant virus attenuates the virulence of ASFV, providing guidance for the development of safer and more reliable attenuated recombinant ASFV vaccines [66].

### 4.4. Inhibition of Apoptosis

Apoptosis, also known as programmed cell death, is a fundamental biological process in multicellular organisms. It plays a critical role in maintaining tissue homeostasis by eliminating unwanted, damaged, or infected cells without inducing an inflammatory response [69]. Apoptosis is tightly regulated by a balance of proapoptotic and antiapoptotic factors [70].

**Figure 2:**
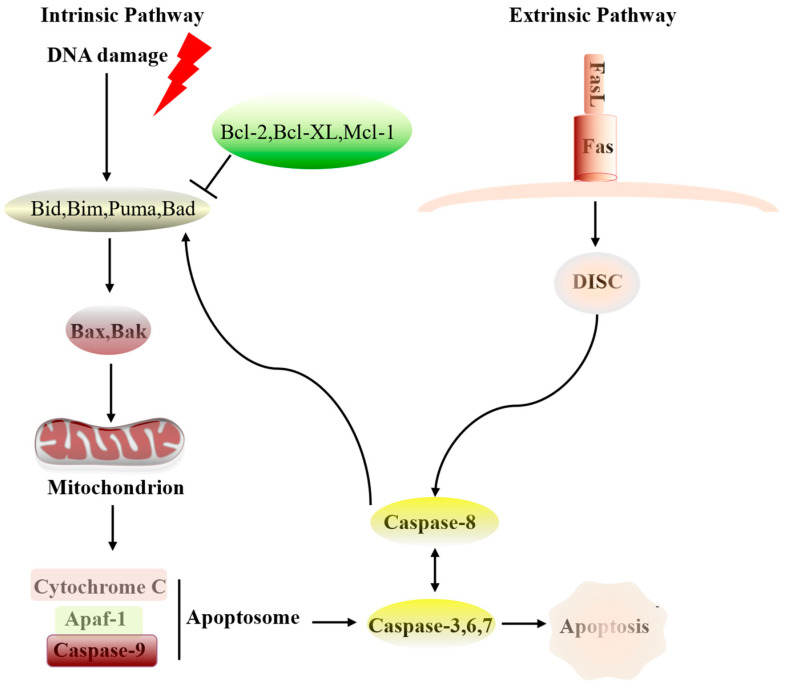
The molecular mechanisms of apoptosis, including intrinsic and extrinsic pathways. Even though these two pathways have distinct starting points, their processes interact with each other and ultimately converge to induce a common apoptotic response.

Proteins of the Bcl-2 family regulate mitochondrial outer membrane permeabilization and play crucial roles in cellular apoptosis pathways, where they can be categorized into two main groups: antiapoptotic members such as Bcl-2, Bcl-XL, Bcl-W, Mcl-1, and CED9; and proapoptotic members, including Bax, Bak, Bcl-XS, Bad, Bik, and Bid [52] (Figure 2). The A179L protein, with a molecular weight of approximately 18 kDa and consisting of 179 amino acids, belongs to the Bcl-2 family and possesses conserved structural domains BH1, BH2, BH3, and BH4, while lacking the corresponding transmembrane domain [71]. The A179L is expressed in both early and late stages of viral infection of ASFV and its sequence shows high conservation across different ASFV strains. Previous studies have shown that the A179L can suppress cell apoptosis induced by p68 in HeLa, as well as apoptosis induced by large molecules in BSC-40 cells, and interacts with proapoptotic proteins such as Bid p13 and p15, which express only the BH3 domain, inhibiting their activity. The A179L also interacts with other members of the proapoptotic protein family, including Bad, Bmf, Bik, and Bim, forming heterodimers that prevent cells from entering the apoptotic pathway [65]. Additionally, it regulates cellular autophagy by interacting with Beclin-1, modulating starvation-induced autophagosome formation [72].

### 4.5. Other Modulations

Given that the inflammatory responses serve as a potent host antiviral defense mechanism in combating viral infection, ASFV has employed evolved mechanisms to inhibit these defense pathways. The host regulates inflammatory responses by controlling the production of proinflammatory factors, inflammatory mediators, and proteins generated in the downstream cascades of the NF-κB. The NLRP3 inflammasome is a cytoplasmic multiprotein complex that assembles and triggers inflammatory immune responses upon exposure to pathogens and tissue damage; predominantly found in macrophages, it comprises NLRP3 protein, ASC adaptor protein, and pro-Caspase-1. Activation of NLRP3 inflammasome-induced IL-1β and pyroptosis requires two priming signals: (1) NF-κB activation, stimulating factors like LPS or viruses to activate NF-κB, thereby inducing upregulation of proinflammatory cytokines and transcriptional levels of NLRP3, pro-IL-1β, etc.; (2) NLRP3 activation, where external pathogens induce assembly of the NLRP3 inflammasome complex and trigger self-cleavage activation of pro-Caspase-1, ultimately leading to maturation of IL-1β within cells and formation of pores on the cell membrane by N-GSDMD, resulting in pyroptosis [36]. ASFV-C84L significantly promotes NLRP3-mediated Caspase-1 activation and expression of proinflammatory cytokines, thus aiding in initiating the inflammatory response.

Ubiquitination is a process where ubiquitin molecules are attached to specific proteins after they have been synthesized [44]. This modification of the ubiquitination serves various purposes, such as directing proteins to different parts within the cell, altering their functions, promoting or inhibiting interactions with other proteins, and labeling them for degradation through the proteasome [73]. ASFV pI215L triggers the interaction between RNF138 and RNF128. This interaction results in an increase in RNF138 levels, causing it to break down RNF128. As a result, the K63-linked ubiquitination of tank-binding kinase 1 (TBK1) is inhibited. Thereby, the downstream of TBK1 is negatively regulated [74]. The immunosuppression protein S273R of ASFV interacts with STAT2 and recruits the E3 ubiquitin ligase DCS1, leading to K48-linked polyubiquitin chains at K55 of STAT2, subsequently prompting the degradation of STAT2 via the proteasome pathway [75]. The ubiquitination serves as a modification mechanism utilized by various viruses, which influences the normal functions of signaling pathways by interacting with specific proteins.

Autophagy is a highly conserved cellular process that involves the degradation and recycling of damaged organelles, misfolded proteins, and other cellular components. The process of autophagy is tightly regulated and involves the formation of double-membrane vesicles called autophagosomes that engulf cytoplasmic cargo targeted for degradation. These autophagosomes then fuse with lysosomes to form autolysosomes, where the cargo is broken down by lysosomal enzymes. It plays a critical role in various physiological processes. The early expressed MGF505-7R exhibits diverse inhibitory actions on STIN-dependent antiviral reaction. It is capable of facilitating the proteasome-mediated degradation of TBK1, as well as the degradation of caspase, IRF7 via autophagosome pathways, and STING through autophagosome-dependent mechanisms. Studies have identified that MGF505-7R enhances the levels of the autophagy-related protein ULK1, leading to the degradation of STING. Additionally, it suppresses IFN-γ-induced signaling pathways mediated by JAK1 and JAK2 [76,77,78].

## 5. Conclusions and Perspective

The immune modulation of ASFV on the host organism consists of manipulating the host cell’s innate immune responses through various encoded proteins. Additionally, ASFV impacts lymphocyte development by hindering antigen presentation and modulating chemokine expression to prevent adaptive immunity activation. Indeed, various structural proteins encoded by ASFV play crucial roles in virus attachment, entry, and replication. In particular, MGF proteins are essential at multiple stages of virus infection in host cells, including transcription, translation, virulence, and immune evasion. For example, MGF-360-10L was identified to facilitate the ubiquitination of JAK1 by recruiting HERC5. MGF-360-9L binds to and degrades STAT1 and STAT2, leading to the suppression of IFN-β signaling and protein degradation. pMGF505-7R inhibits NF-κB activity by binding to IKK, blocking its translocation to the nucleus. MGF360-13L inhibits the activation of the cGAS-STING-mediated IFN signaling pathway. However, our current understanding of the functions and characteristics of ASFV proteins are not fully understood, and the complex and diverse immune regulation mechanisms after invading the host remain unclear. Therefore, a significant effort is required to gain a clear understanding of the specific functions of over 150 nonstructural proteins and structural proteins.

In conclusion, the intricate network of pathways and distinct stages involved in immune modulation poses challenges for the development of effective vaccines and treatments, particularly due to issues regarding safety and stability. These obstacles stem from the multifaceted nature of the process, which includes limited comprehension of virus–host interactions and the complex structure of the ASFV itself. While immunization with high-dose inactivated ASFV or γ-irradiated ASFV is considered safe, the immune protection efficacy remains suboptimal. Meanwhile, the nucleic acid vaccines, subunit vaccines, and viral vector vaccines only offer partial immunity. Therefore, studies on the functions of ASFV-encoded proteins, particularly the proteins mediating ASFV immune escape and immunosuppression, are very important for the vaccine development and design. Attenuated ASFV strains derived from either naturally weakened variants or genetically engineered constructs can confer full protection upon immunized pigs. However, viral presence persists in blood and certain organs, leading to intermittent shedding and posing a risk of horizontal transmission (Table 2).

Through modern virology and immunology techniques, identifying the ASFV immunomodulatory genes and subsequently discovering and characterizing essential virulence genes of ASFV becomes imperative. This will elucidate their roles in virus infection, immune evasion, and pathogenesis. Utilizing techniques such as CRISPR, genomics, transcriptomics, proteomics, and metabolomics can help unravel the specific molecular mechanisms underlying the ASFV lifecycle or immune modulation. This review summarized the ASFV virulence-associated genes and proteins while providing an overview of the current research on how ASFV suppresses the innate and adaptive immune responses in the host by its different proteins. It points out areas of our knowledge of how ASFV impacts the cellular and humoral immune systems of the host. The process of immunosuppression of ASFV is characterized by diversity and complexity and more mechanisms underlying the interaction between ASFV and the host await discovery. By combining classical molecular biology research methods, critical replication or immune suppression mechanisms of ASFV can be further identified. This could provide theoretic support for the discovery of suitable drugs or the design of stable and safe vaccines to make ASF treatable, controllable, and preventable.

## Figures and Tables

**Table 1:** Immunosuppression proteins of ASFV.

Viral Protein	Function	References
pC129R	Target Cyclic GMP-AMP To Inhibit the cGAS-STING Signaling Pathway	[25]
P17	Inhibits cGAS-STING signaling pathway through interacting with STING	[26]
pM1249L	Suppress phosphorylation of TANK-binding kinase (TBK) 1 and degrading IFN regulatory factor 3 (IRF3)	[27]
pMGF505-11R	Binds to STING and promotes its degradation through the lysosomal and autophagy mechanisms	[28]
pMGF360-11L	Inhibits IL-1, IL-6, and IFN-β secretion	[29]
pA528R	Inhibits phosphorylation of p65	[30]
pEP153R	Prevent apoptosis via activating the p53 and caspase 3 pathways	[31]
DP71L	Inhibits apoptosis at early infection	[32]
pE199L	Promotes cell autophagy through the interaction of PYCR2	[33]
pA137R	Inhibited the nuclear import of IRF3	[34]
pI215L	Encode the ubiquitin-conjugating enzyme making	[35]
P54, pA179L	Regulate the programmed cell death	[36]
CD2v	Hinder lymphocyte proliferation	[25]

**Table 2:** The disadvantages and targets of various vaccines.

Type of Vaccine	Disadvantage	Advantage and Target
Traditional vaccine	Cannot provide strong immunity and has poor long-term immunogenicity.	The simple preparation process, high safety.
Subunit vaccine	Cannot effectively induce the body to generate a cellular immune response.	Immunize animals with an adjuvant, high safety.
Nucleic vaccine	Can induce the body to generate a cellular immune response	The spectrum of antigens is not broad enough.
Live attenuated vaccine	The risk of increased virulence and the potential for recombination between different strains, resulting in the emergence of highly virulent strains.	Avoiding key issues associated with inactivated vaccines, subunit vaccines, and DNA vaccines, it can replicate continuously in the animal body, mimicking natural infection pathways to effectively induce both humoral and cellular immune responses. Additionally, it does not require adjuvants to enhance the immune response.

## Abbreviations

The following abbreviations are used in this manuscript:

ASFV	African swine fever virus
ASF	African swine fever
VF	virus factory
APCs	Antigen-presenting cells
MGF	multigene family
TBK	TANK-binding kinase
IRF	IFN regulatory factor
PAMP	Pathogen-associated molecular patterns
PRR	Pattern recognition receptors
NOD	Nucleotide oligomerization domain
MHC	major histocompatibility complex
JAK	Janus Kinase
STAT	Signal Transducers and Activators of Transcription
cGAMP	cyclic GMP-AMP
ISGF	IFN-stimulated gene factor
ISRE	IFN-stimulated response element
ISGs	IFN-stimulated genes
IKK	IκB kinase
IKKβ	IκB kinase β
TYK2	tyrosine kinase2
ISGF	Interferon-Stimulated Gene Factor
